# Orientin Reduces Myocardial Infarction Size via eNOS/NO Signaling and Thus Mitigates Adverse Cardiac Remodeling

**DOI:** 10.3389/fphar.2017.00926

**Published:** 2017-12-21

**Authors:** Fangfang Li, Jing Zong, Hao Zhang, Peijie Zhang, Luhong Xu, Kai Liang, Lu Yang, Hui Yong, Wenhao Qian

**Affiliations:** ^1^Department of Cardiology, The Affiliated Hospital of Xuzhou Medical University, Xuzhou, China; ^2^Institute of Cardiovascular Disease Research, Xuzhou Medical University, Xuzhou, China; ^3^Emergency Department, The Affiliated Hospital of Xuzhou Medical University, Xuzhou, China

**Keywords:** orientin, myocardial infarction, cardiac remodeling, eNOS, nitric oxide, reactive oxygen species

## Abstract

Orientin is a flavonoid extracted from Chinese traditional herb, Polygonum orientale L. Previous study has reported that orientin protected myocardial from ischemia reperfusion injury. However, whether orientin could protect against cardiac remodeling after myocardial injury remains unclear. The aim of our study is to investigate the effects of orientin in the progression of cardiac remodeling after myocardial infarction (MI). Mice cardiac remodeling model was established by left coronary artery ligation surgery. Experimental groups were as follows: vehicle-sham, orientin-sham, vehicle-MI, and orientin-MI. Animals were treated with vehicle or orientin (40 mg/kg) for 25 days starting 3 days after surgery. After 4 weeks of MI, mice with orientin treatment had decreased mortality and improved cardiac function. Significantly, at 4 weeks post-MI, orientin treatment decreased fibrosis, inflammatory response, and cardiomyocyte apoptosis. Furthermore, orientin treatment attenuated the hypoxia-induced neonatal rat cardiomyocyte apoptosis and increased cell viability. Additionally, orientin supplementation mitigated oxidative stress in remodeling heart tissue and cardiomyocytes exposed to hypoxia as measured by 2′,7′-dichlorodihydrofluorescein diacetate fluorescent probe. Mechanistically, orientin promotes cardioprotection by activating the eNOS/NO signaling cascades, which was confirmed by eNOS inhibitor (L-NAME) *in vitro* and *in vivo*. Inhibition of oxidative stress by orientin via eNOS/NO signaling cascades in the heart may represent a potential therapy for cardiac remodeling.

## Introduction

The heart undergoes a series of cardiac wound healing responses following MI injury. The cardiomyocyte necrosis and apoptosis causes a series of molecular and cellular remodeling, including inflammatory response, cardiac hypertrophy, and fibrosis (Santos-Gallego et al., 2014; Bhatt et al., 2017). In the first case, these changes of the heart exert a compensatory effect, maintaining normal cardiac function (Schirone et al., 2017). In contrast, after sustained stress, cardiac remodeling leads to a progressive and irreversible dysfunction of the heart, and thus results in the development of chronic heart failure and death (Prabhu and Frangogiannis, 2016). During the cardiac remodeling process, oxidative stress plays a vital role in the transition of heart disease to heart failure (Jain et al., 2015). Excess oxidative stress leads to several cytotoxicities, such as lipid peroxidation, protein oxidation, and DNA damage, which cause changes in calcium-transport proteins and the activation of hypertrophy signaling pathways, triggering cardiomyocyte dysfunction and apoptosis, and fibroblast proliferation (Azevedo et al., 2016; Wu et al., 2017). Therefore, targeting oxidative stress seems to be a potential strategy for the treatment of cardiac remodeling.

Orientin is a flavonoid component isolated from natural plants, such as Ocimum sanctum, phyllostachys species (bamboo leaves) (Xiao et al., 2017). Over the past decade, orientin has been suggested to possess abundant properties, such as antioxidant, antiviral, anti-inflammatory (Yoo et al., 2014), anti-glycation, anti-cancer, and anti-thrombus activities (Wang et al., 2017; Xiao et al., 2017). Wang X reported that orientin protects against cerebral ischemia/reperfusion injury by regulating TLR4/NF-κB/ TNF-α signaling in rat (Wang et al., 2017). Orientin was observed to protect heart I/R injury, and cardiomyocyte H/R injury (Fu et al., 2006; Lu et al., 2012) by regulating autophagy (Liu et al., 2016). Despite definite anti- I/R and H/R injury effect, the anti-cardiac remodeling effects of orientin have not been fully elucidated to date. Accordingly, in the current study, we sought to evaluate whether orientin MI induced cardiac remodeling.

## Materials and Methods

### Animals

All animal care and experiments were performed according to the Guidelines for the Care and Use of Laboratory Animals published by the National Institutes of Health (United States) (NIH Publication, revised 2011) and the institutional guidelines of the Animal Care and Use Committee of Xuzhou Medical University (Xuzhou, China; Approval number: JSXZ-2015-1223-012; Approval data: 05/12/2015). Animals were housed as previous described (Yuan et al., 2014). We purchased 8–10-weeks-old male C57BL/6 mice (body weight: 25.5 ± 2 g) from the Institute of Laboratory Animal Science, Chinese Academy of Medical Sciences (Beijing, China). Shanghai Winherb Medical Science, Co. Ltd. (Shanghai, China) provided the purified orientin (>98%). The left coronary artery ligation was performed in a blinded manner for all groups, according to a previous study (Bao et al., 2015). The animals were divided into eight groups randomly: vehicle-sham (*n* = 15), Orientin-sham (*n* = 15), vehicle-MI (*n* = 20), and Orientin-MI (*n* = 20); L-NAME-sham (*n* = 15), Orientin+L-NAME-sham (*n* = 15), L-NAME-MI (*n* = 20), and Orientin+L-NAME-MI (*n* = 20). Three days after MI or sham procedure, mice were treated with orientin (dissolved in normal saline) or vehicle (the same volume of normal saline) for 25 days by oral gavage, and the dose of orientin was 40 mg/kg. L-NAME was subjected in the drinking water (2 mg/mL) together with orientin administration.

### Echocardiography and Hemodynamic Evaluation

Echocardiography and hemodynamic measurement was performed according to our previous description (Wu et al., 2015). The left ventricle (LV) end-systolic diameter (LVESd), LVEDd, LVEF, and FS were analyzed. For the hemodynamic analysis, dp/dtmax, dp/dtmin were analyzed.

### Cardiac Morphology and Histomorphometric Analysis

Hematoxylin–eosin (H&E) and PSR staining was performed according to our previous description (Wu et al., 2015). The quantitative digital analysis system (NIH Image 1.6, National Institutes of Health, United States) was used to analyzed the infarct size, which was expressed as a percentage of the total LV area. The software, Image-Pro Plus 6.0, was used to analyzed interstitial collagen deposition by PSR staining. Immunofluorescence staining of Wheat Hemagglutinin (1:100, Invitrogen, United States) was used to detect cardiomyocytes size as previous study described (Santos-Gallego et al., 2016).

For immunohistochemistry staining, hearts were incubated with anti-CD68 (1:100), anti-TNFa (1:100), or anti-4-hydroxynonenal (4-HNE, 1:100) (Abcam, Cambridge, MA, United States) followed by incubation with anti-rabbit HRP reagent (Gene Tech, Shanghai, China) and a peroxide-based substrate DAB kit (Gene Tech, Shanghai, China). Light microscopy (H550L, Tokyo, Japan) was used for analysis.

Apoptosis Detection Kit (Millipore, Temecula, CA, United States) was used to detect apoptosis according to the manufacturer’s instructions. Briefly, hearts were incubated with HRP-labeled dUTP followed by a peroxide-based substrate DAB kit. Microscopy (BX51, Olympus, Japan) was used to analyzed the apoptosis-positive cells.

### Neonatal Rat Cardiomyocyte (NRCM) Culture and Treatment

Primary NRCMs were isolated and cultured according to previous study (Wu et al., 2016). NRCMs were cultured with serum-free DMEM/F12 for 12 h before pretreatment with orientin (1, 5, 10, 30 μM) or NAC (2 mM, Sigma), L-NAME (100 μM, Sigma), L-VINO (10 μM, Santa Cruz), or L-Canavanine (1 mM) for 12 h. Next, cells were exposed to hypoxia for 24 h in a Biospherix C-Chamber (model C-274, Biospherix, Redfield, NY, United States), inside a standard culture chamber according to a previous study (Bao et al., 2015). The cell viability was measured by a cell counting kit-8 assay (CCK8, CK04, Donjindo, Tokyo, Japan).

### Oxidative Stress

Reactive oxygen species was detected using the 2′,7′-dichlorodihydrofluorescein diacetate fluorescent probe (Beyotime, Haimen, China) according to the manufacturer’s protocols with a luminometer (Synergy HT, BioTek, United States) by detecting fluorescence intensity at 488 nm excitation wavelength and 525 nm emission wavelength.

Lysate from fresh mice hearts and cardiomyocytes was collected. Commercial kits (Beyotime, Haimen, China) were used to detect the total SOD activity and NADPH oxidase activity as well as the level of lipid peroxidation.

### NO Level

Nitric oxide level was determined as the measurement of nitrate plus nitrite using the Griess reaction assay (Cayman Chemical, Ann Arbor, MI, United States) according to the manufacturer’s manual (Niu et al., 2012).

### Annexin V Staining

Cell apoptosis was detected by annexin V staining according to the manufacturer’s protocols (Beyotime, Haimen, China). Briefly, after being washed three times with PBS, cells were digested and incubated with annexin V for 30 min. A fluorescence microscope was used to observe and analyze the result.

### Quantitative Real-Time PCR and Western Blot Analysis

Total RNA was extracted from hearts and cultured NRCMs in TRIzol reagent (Invitrogen, Carlsbad, CA, United States), 2 μg of total RNA was reverse-transcripted to strand cDNA with oligo (dT) primers and a cDNA synthesis kit (Roche, Mannheim, Germany). A LightCycler 480 SYBR Green Master Mix (Roche, Mannheim, Germany) was used to quantify the mRNA expression level. All mRNA expression was normalized to GAPDH mRNA levels. **Table 1** shows the primers utilized for amplification.

**Table 1:** Primer sequences used for RT-PCR.

mRNA	Forward	Reverse
Collagen I	AGGCTTCAGTGGTTTGGATG	CACCAACAGCACCATCGTTA
Collagen III	AAGGCTGCAAGATGGATGCT	GTGCTTACGTGGGACAGTCA
CTGF	AGGGCCTCTTCTGCGATTTC	CTTTGGAAGGACTCACCGCT
TGFβ	ATCCTGTCCAAACTAAGGCTCG	ACCTCTTTAGCATAGTAGTCCGC
IL-1	CCGTGGACCTTCCAGGATGA	GGGAACGTCACACACCAGCA
IL-6	AGTTGCCTTCTTGGGACTGA	TCCACGATTTCCCAGAGAAC
TNFα	ACTGAACTTCGGGGTGATCGGT	TGGTTTGCTACGACGTGGGCTA
GAPDH	ACTCCACTCACGGCAAATTC	TCTCCATGGTGGTGAAGACA

*Sequences are listed 5′–3′.*

Total proteins were extracted from LV tissues and cultured NRCMs with RIPA lysis buffer. A Pierce BCA Protein Assay Kit (23225, Thermo Scientific, MIT, United States) was used to measure protein concentration. Next, 50 μg of protein was loaded for electrophoresis in an SDS-PAGE gel followed by transfer to a PVDF membrane (IPVH00010, Millipore, Billerica, MA, United States). The following primary antibodies were used: rabbit anti-c-caspase-3, rabbit ani-Bax, anti-Bcl-2, anti-nNOS (Cell Signaling Technology, Inc., Danvers, MA, United States), rabbit anti-iNOS, rabbit anti-eNOS (P-S1177), rabbit anti-GAPDH monoclonal antibody (Abcam, Cambridge, MA, United States), rabbit anti-eNOS (Santa Cruz Biotechnology, Santa Cruz, CA, United States). After incubation with the second antibody, a two-color infrared imaging system (Odyssey; LI-COR Biosciences, Lincoln, NE, United States) was used to scan membranes. Each sample was normalized against GAPDH protein levels.

### Statistical Analysis

Data are presented as mean ± standard deviation (SD). A one-way analysis of variance (ANOVA) followed by Tukey’s *post hoc* test was used to analyze differences among groups. Student’s *t*-test was used to analyze comparisons between two groups. A *p*-value < 0.05 is considered to be significant.

## Results

### Orientin Ameliorates Post-infarction Outcomes of MI

**FIGURE 1:**
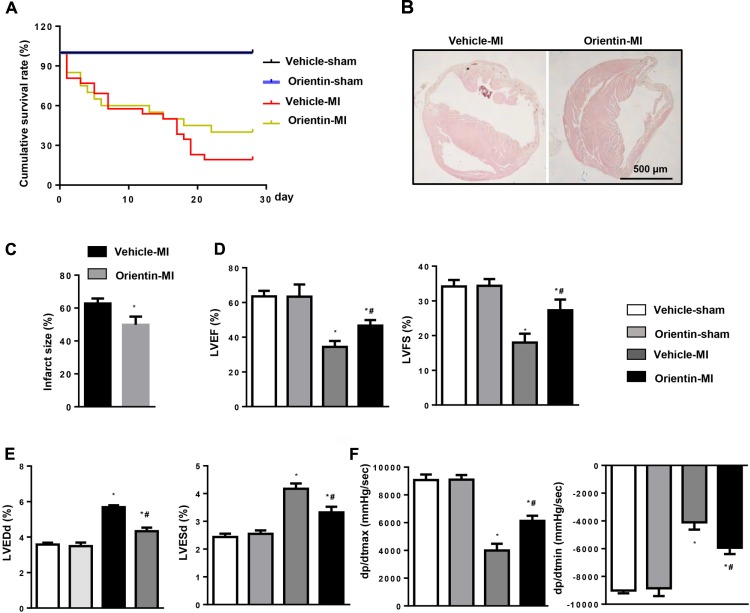
Orientin ameliorates post-infarction outcomes of MI. **(A)** Kaplan–Meier survival analysis of mice in vehicle-MI and orientin-MI group in the first 4 weeks after MI. H&E staining of mice heart in vehicle-MI and orientin-MI group 4 weeks after MI (**B**, representative image; **C**, quantitation result); ^∗^*p* < 0.05 vs. vehicle-MI. Echocardiographic **(D,E)** and hemodynamic **(F)** results for mice in the four groups at 4 weeks post-MI (*n* = 6–8); ^∗^*p* < 0.05 vs. vehicle-sham; ^#^*p* < 0.05 vs. vehicle-MI.

Orientin did not affect cardiac structure and function during basal conditions (**Figure 1**). No mice underwent death in the sham groups during the observation period (**Figure 1A**). However, during the 2 weeks after MI, the survival rate increased, to reveal the same in both vehicle group and orientin-treated group, due to cardiac rupture, which caused a high rate of sudden death. However, up to 4 weeks after MI, compared with vehicle treated group, the survival rate was higher in the orientin-treated group (**Figure 1A**). Additionally, the infarct size at 4 weeks after MI was significantly smaller in orientin-treated mice hearts than in vehicle controls, as shown by H&E staining (**Figures 1B,C**). Furthermore, MI-induced cardiac dysfunction was strikingly improved by orientin with higher LVEF, and LVFS, increased dP/dtmax, dP/dtmin, and reduced LVEDd, LVESd than those in vehicle mice, as accessed by echocardiography and hemodynamic measurements (**Figures 1D–F**).

### Orientin Attenuates MI-Induced Hypertrophy, Fibrosis, and Inflammatory Response

**FIGURE 2:**
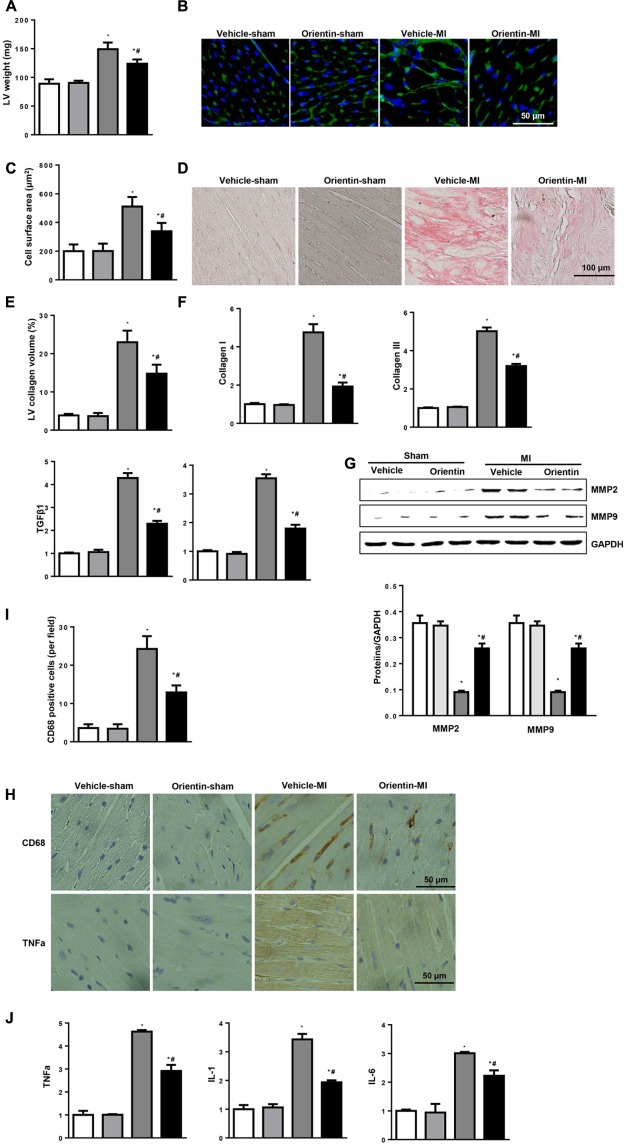
Orientin attenuates MI-induced fibrosis and inflammatory response. **(A)** LV weight in the indicated mice 4 weeks after MI (*n* = 6–8). **(B)** Immunofluorescent staining of wheat hemagglutinin (WGA) (*n* = 6). **(C)** Analysis of cell surface area (*n* > 200 cell per group). **(D)** PSR staining of heart to analyze the LV collagen volume (*n* = 25+ fields per experimental group). **(E)** Statistical analysis of the CSA and LV collagen volume (%). **(F)** The relative mRNA levels of collagen I, collagen III, TGFβ1, and CTGF, in LV samples from vehicle and orientin-treated mice heart (*n* = 6). **(G)** Representative Western blots and quantitation of MMP2, MMP9 in the heart tissue of vehicle and orientin-treated mice at 4 weeks after sham or MI surgery (*n* = 6). **(H)** Immunohistochemistry staining showing the number of CD68-positive cells and TNFα release in the heart cross-sections of the vehicle and orientin-treated mice. **(I)** Statistical analysis of the number of CD68-positive cells in the indicated group (*n* = 6). **(J)** The relative mRNA levels of TNFα, IL-1 and IL-6 in samples from vehicle and orientin-treated mice heart (*n* = 6); ^∗^*p* < 0.05 vs. vehicle-sham; ^#^*p* < 0.05 vs. vehicle-MI.

Pathological cardiac hypertrophy, fibrosis, and inflammatory response are the major features in post-MI cardiac remodeling that are associated with heart failure (Wu et al., 2017). Therefore, cardiac hypertrophy, fibrosis, and inflammatory response were determined. The increased LV weight 4 weeks after MI was decreased by orientin treatment (**Figure 2A**) as well as the cell surface area assessed by WGA staining (**Figures 2B,C**). A dramatic interstitial fibrosis was observed in vehicle-MI mice hearts by PSR staining, while compared with the vehicle control group, orientin treatment reduced the collagen volume. Additionally, compared with vehicle mice at 4 weeks after MI, the mRNA levels of fibrotic markers (collagen I, collagen III and CTGF and TGFβ) were much lower in orientin-treated mice (**Figures 2D–F**). Orientin also decreased the MMP2 and MMP9 expression 4 weeks after MI (**Figure 2G**). Orientin may attenuate ECM remodeling through decreasing collagen accumulation and reducing MMP activity. Coincident with the ameliorated cardiac fibrosis, the inflammatory response was also inhibited by orientin as assessed by decreased CD68-labeled macrophage infiltration and reduced pro-inflammatory cytokine expression (TNFα, IL-1, IL-6) (**Figures 2H–J**).

### Orientin Inhibits MI-Induced Apoptosis

**FIGURE 3:**
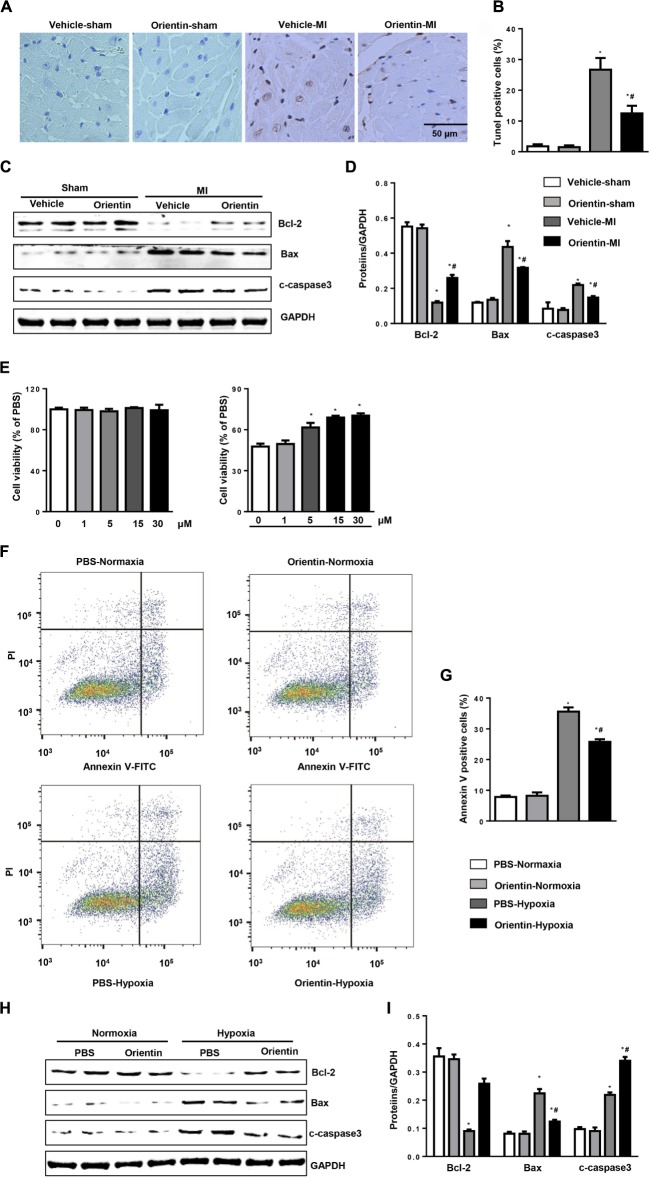
Orientin inhibits MI-induced apoptosis. TUNEL staining **(A)** and quantitation **(B)** in the hearts of vehicle and orientin-treated mice at 4 weeks post-MI (*n* = 6). **(C,D)** Representative Western blots and quantitation of Bax, Bcl-2, and C-caspase3 in the heart tissue of vehicle and orientin-treated mice at 4 weeks after sham or MI surgery (*n* = 6); ^∗^*p* < 0.05 vs. vehicle-sham; ^#^*p* < 0.05 vs. vehicle-MI. **(E–I)** NRCMs were pretreated with orientin (1, 5, 15, 30 μM) for 12 h, then exposed to hypoxia for 24 h. **(E)** MTT assays were performed to detect cell viability (*n* = 5, ^∗^*p* < 0.05 vs. hypoxia-0 μM group). Annexin V staining **(F)** and quantitation **(G)** in the indicated group (*n* = 6). Representative Western blots **(H)** and quantitation **(I)** of Bax, Bcl-2, and C-caspase3 in the indicated group (*n* = 6); ^∗^*p* < 0.05 vs. vehicle-normoxia; ^#^*p* < 0.05 vs. vehicle-hypoxia.

TUNEL staining was used to detect the extent of apoptosis on the peri-infarct heart tissues. Compared with sham heart, MI induced significant cell apoptosis after 4 weeks of remodeling process. In contrast, compared with vehicle-treated mice hearts, orientin treatment caused reduced TUNEL-positive nuclei (**Figures 3A,B**). Western blot was analyzed to detect the apoptosis-associated protein expression (Bax, Bcl-2, and cleaved caspase 3). MI induced alternation of apoptosis-associated protein expression, which caused an increased ratio of Bax/Bcl-2, triggering cell apoptosis. Conversely, compared with vehicle control, orientin treatment decreased the expressions of pro-apoptosis proteins Bax and cleaved caspase 3, and increased the expression of the anti-apoptosis protein Bcl-2 in hearts 4 weeks post-MI (**Figures 3C,D**).

The effect of orientin on cardiomyocyte was investigated *in vitro* study. Cultured NRCMs were pretreated with orientin (0, 1, 5, 15, 30 μM) and exposed to hypoxia for 24 h. Our *in vitro* data showed that four concentrations of orientin did not affect the viability of NRCMs (**Figure 3E**). Consistent with the *in vivo* results, orientin (30 μM) decreased the number of annexin V-positive cardiomyocytes induced by hypoxia (**Figures 3F,G**) and increased cell viability (**Figure 3E**). Accordingly, the expressions of Bax and cleaved caspase 3 were markedly decreased and Bcl-2 was significantly enhanced after orientin pretreatment (**Figures 3H,I**).

### Orientin Ameliorates MI-Induced Oxidative Stress

**FIGURE 4:**
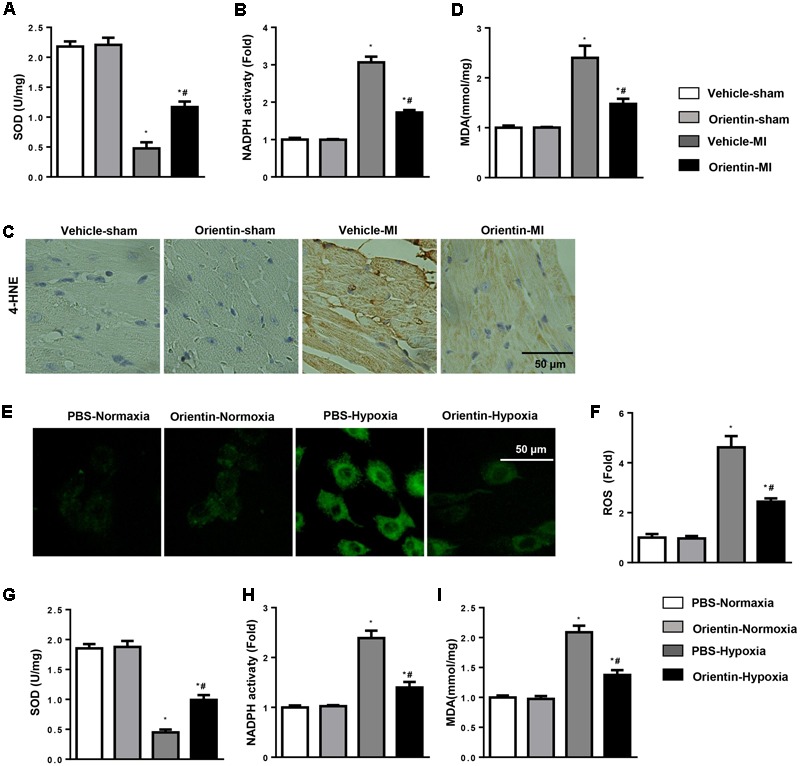
Orientin ameliorates MI-induced oxidative stress. SOD **(A)** and NADPH oxidase **(B)** activity in the heart tissue of vehicle and orientin-treated mice (*n* = 6). **(C)** Immunohistochemistry staining of 4-hydroxynonenal (4-HNE) in the heart cross-sections of the vehicle and orientin-treated mice (*n* = 6). **(D)** The levels of lipid oxidation end products, malondialdehyde (MDA) in the indicated mice heart. **(E–I)** NRCMs were pretreated with orientin (1, 5, 15, 30 μM) for 12 h, then exposed to hypoxia for 24 h. The ROS was detected and quantified by DCFH-DA with light microscopy **(E)**, ELISA reader **(F)**. The levels of SOD **(G)**, NADPH oxidase **(H)** activity, and malondialdehyde **(I)** in the indicated group (*n* = 6); ^∗^*p* < 0.05 vs. vehicle-normoxia; ^#^*p* < 0.05 vs. vehicle-hypoxia.

Increased oxidative stress aggravated cardiac remodeling. Compared with the sham group, 4 weeks after MI, the remodeling heart revealed a decreased SOD activity and enhanced NADPH oxidase activity. Compared with vehicle-treated group, orientin treatment markedly improved this impaired balance in cardiac tissues (**Figures 4A,B**). The increased production of myocardial lipid peroxidation in remodeling heart was also reduced by orientin (**Figure 4C**), which was also confirmed by immunohistochemical analyses of 4-HNE (**Figure 4D**).

The antioxidative stress effect of orientin was also investigated in NRCMs. As expected, orientin (30 μM) significantly decreased the ROS generation, enhanced the SOD activity, and reduced NADPH oxidase activity and lipid peroxidation in NRCMs exposed to hypoxia (**Figures 4E–I**).

### Orientin Enhances eNOS Activation and NO Production

**FIGURE 5:**
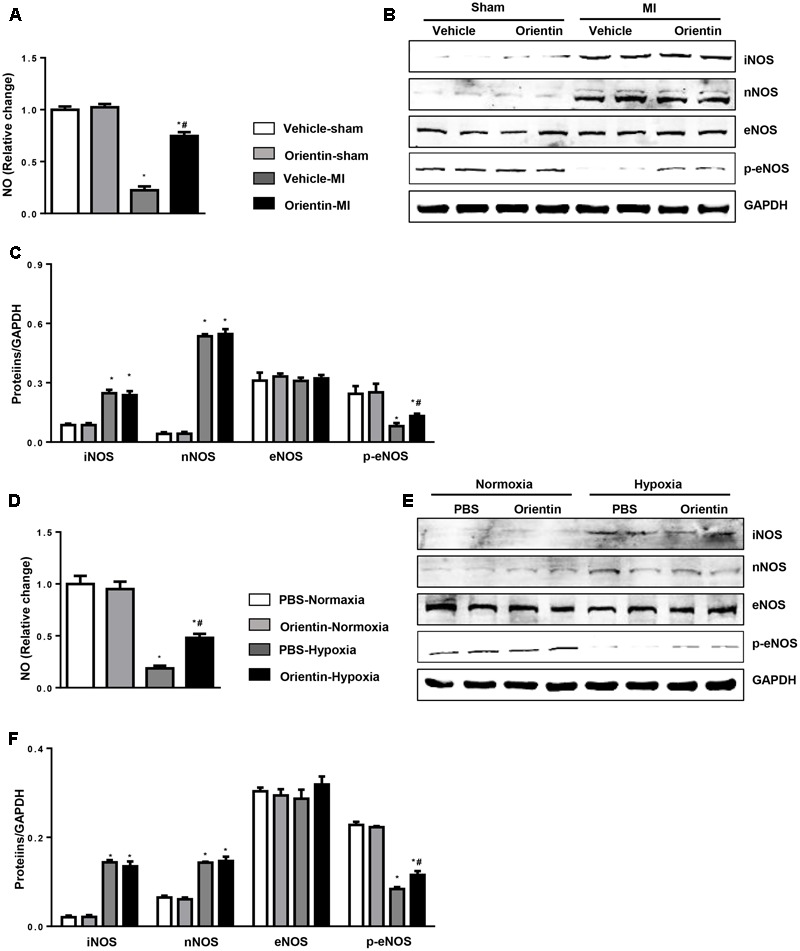
Orientin enhances eNOS activation and NO production. **(A)** NO production in the heart tissue of vehicle and orientin-treated mice at 4 weeks after sham or MI surgery (*n* = 6). Representative Western blots **(B)** and quantitation **(C)** of iNOS, nNOS, eNOS, and phosphorylated (p-) eNOS in the heart tissue of vehicle and orientin-treated mice at 4 weeks after sham or MI surgery (*n* = 6, ^∗^*p* < 0.05 vs. vehicle-sham; ^#^*p* < 0.05 vs. vehicle-MI). **(D)** NO production in the orientin (30 μM) pretreated NRCMs after exposure to hypoxia for 24 h (*n* = 6). Representative Western blots **(E)** and quantitation **(F)** of iNOS, nNOS, eNOS, and phosphorylated (p-) eNOS in the orientin (30 μM) pretreated NRCMs after exposure to hypoxia for 24 h (*n* = 6); ^∗^*p* < 0.05 vs. vehicle-normoxia; ^#^*p* < 0.05 vs. vehicle-hypoxia.

Nitric oxide signaling has been shown to have a significant role in oxidation–reduction (Tang et al., 2014). Since orientin exerts an antioxidative effect, we detected the NO signaling. The levels of NO were significantly decreased in hearts after MI, but were increased in orientin-treated mice heart (**Figure 5A**). Based on the results above, we next investigated whether orientin affected NOS expression and activation. We found that the iNOS and nNOS were increased after MI, but not affected by orientin. Compared with the sham heart, the total eNOS was not changed in the remodeling heart, but the p-eNOS was decreased in remodeling heart, and orientin increased the phosphorylation of eNOS (**Figures 5B,C**). The same results were obtained in NRCMs (**Figures 5D–F**).

### Protective Effects of Orientin Rely on eNOS *in Vitro*

**FIGURE 6:**
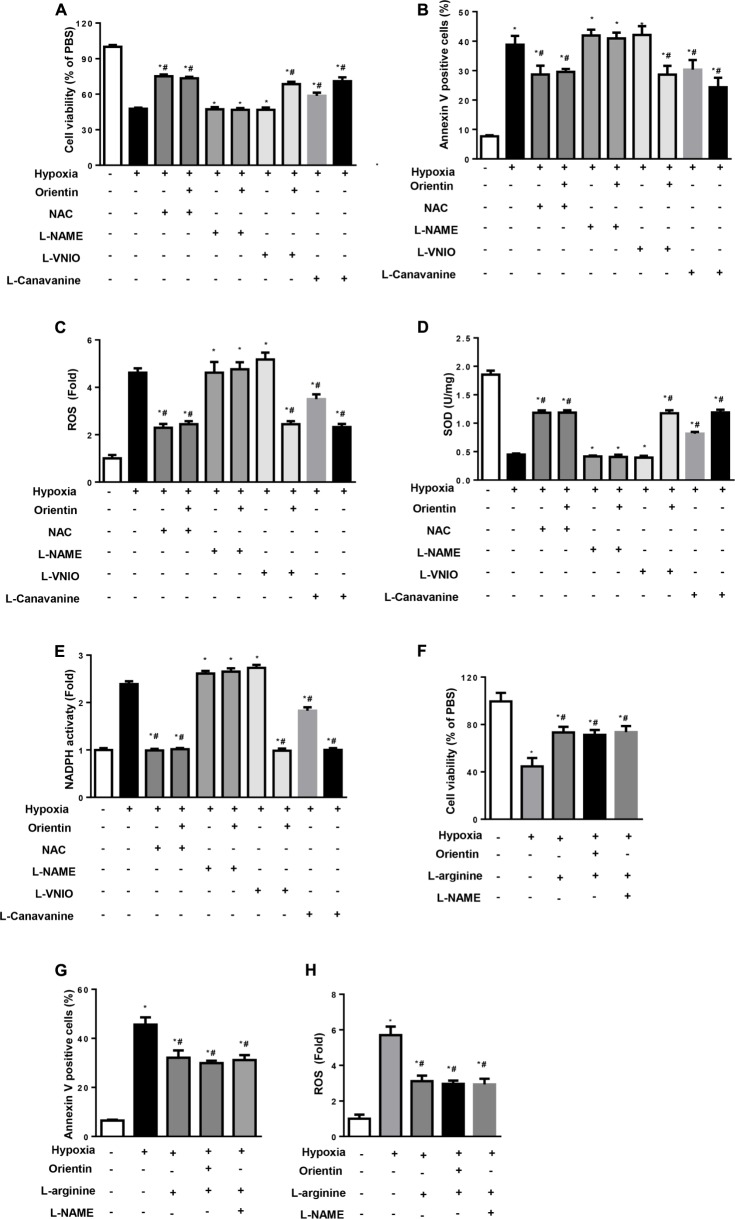
The protective effects of orientin relies on eNOS *in vitro.* NRCMs were pretreated with orientin (30 μM), NAC (2 mM), L-NAME (100 μM), L-VINO (10 μM), or L-Canavanine (1 mM), L-arginine (2 mM) and exposed to hypoxia for 24 h. **(A,F)** MTT detected the cell viability in the indicated group. **(B,G)** Annexin V-positive cells in the indicated group. **(C,H)** The ROS was detected and quantified by DCFH-DA. The levels of SOD **(D)**, NADPH oxidase **(E)** activity in the indicated group (*n* = 6); ^∗^*p* < 0.05 vs. vehicle-normoxia; ^#^*p* < 0.05 vs. vehicle-hypoxia.

The effect of orientin on eNOS/NO signaling in response to hypoxic stimulation was confirmed *in vitro* study. NRCMs were pretreated with NAC, L-NAME (non-specific NOS inhibitor), L-VINO (selective inhibition of nNOS), or L-Canavanine (selective inhibition of iNOS). As a result, NAC exerted the same effects as orientin, and NAC could not affect the protective effects of orientin (**Figures 6A–E**). Interestingly, the non-specific NOS inhibitor, L-NAME, almost abolished the protective effects of orientin on cardiomyocytes exposed to hypoxia, while L-VINO, the selective inhibitor of nNOS, and L-Canavanine, the selective inhibitor of iNOS, could not prevent the orientin-induced anti-apoptosis and antioxidative stress effect (**Figures 6A–E**). To detect whether increased NO mediated the key effects of orientin, L-arginine, the substrate for NO production was used. As a result, L-arginine could mimic the effect of orientin and even reverse the phenotype induced by L-NAME (**Figures 6F–H**). These data suggested that increased NO mediated the key effect of orientin, which attenuated the ROS generation.

### eNOS Blocking Abolishes the Protective Effects of Orientin *in Vivo*

**FIGURE 7:**
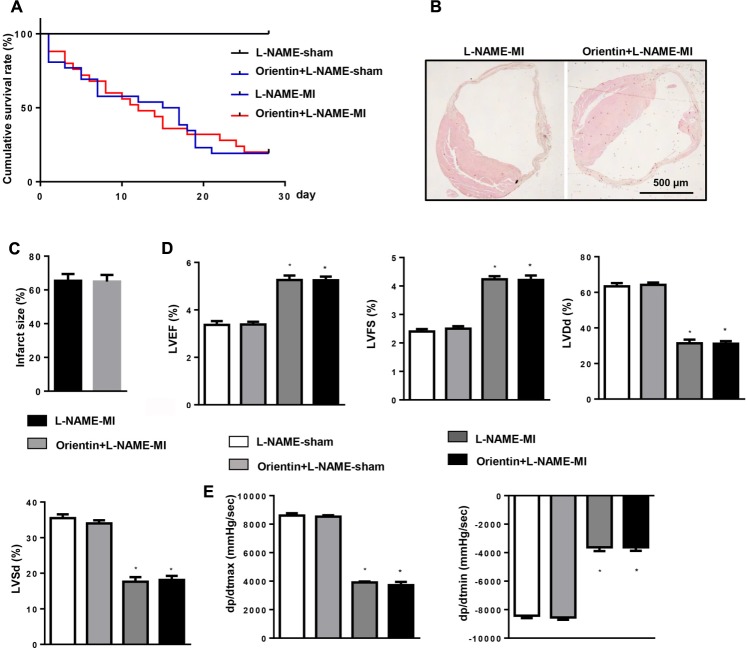
eNOS blocking abolishes the protective effects of orientin *in vivo.* Mice were subjected to MI, then treated with eNOS inhibitor (L-NAME) and orientin for 25 days. **(A)** Kaplan–Meier survival analysis of mice in the indicated group 4 weeks after MI. H&E staining of mice heart in the indicated group after 4 weeks of MI (**B**, representative image; **C**, quantitation result). Echocardiographic **(D)** and hemodynamic **(E)** results for mice in the four groups at 4 weeks post-MI (*n* = 6–8); ^∗^*p* < 0.05 vs. L-NAME-sham; ^#^*p* < 0.05 vs. L-NAME-MI.

The dependence of orientin on eNOS was further detected *in vivo* study. Mice were subjected to MI. After 3 days, orientin and L-NAME were administered. After 4 weeks of MI, we found that the survival rate and infarction area were not improved in the orientin+L-NAME group (**Figures 7A–C**). L-NAME also abolished the cardiac protective effects of orientin, as accessed by the deteriorated cardiac systolic and diastolic function as well as the augmented ventricular cavity (**Figures 7D,E**).

## Discussion

Orientin has been shown to possess abundant properties, such as antioxidant, antiviral, anti-inflammatory (Yoo et al., 2014), anti-glycation, anti-cancer, and anti-thrombus activities (Wang et al., 2017; Xiao et al., 2017). However, the effects of orientin on cardiac remodeling, especially on the remodeling process after MI, are still largely unknown. In the present study, we showed that oral orientin treatment ameliorated cardiac remodeling post-MI. The beneficial effects of orientin were associated with decreased fibrosis and inflammatory response, as well as decreased cardiomyocyte apoptosis. Orientin also normalized the MI-induced disturbances in oxidative stress. Mechanically, orientin suppressed the post-MI remodeling process via augmenting the eNOS/NO signaling.

Multiple factors contribute to the progression of cardiac remodeling and LV dysfunction post-MI (Schirone et al., 2017). Cardiomyocyte death is a crucial event underlying the development of cardiac dysfunction during stress and determining the progression of cardiac abnormalities over time (Wu et al., 2017). Additionally, cardiac hypertrophy and fibrosis and a progressive impairment of contractility and relaxation together orchestrate the detrimental evolution of cardiac remodeling (Azevedo et al., 2016). Several molecular pathways converge in cardiac remodeling. It has been demonstrated that after a cardiac injury, inflammation is sustained through the upregulation of cytokine release, leading to fibroblast proliferation and metalloproteinase activation (Dhalla et al., 2012). Previous study reported that orientin protected ischemia/reperfusion (I/R) heart and cardiomyocytes by inhibition apoptosis (Fu et al., 2006; Lu et al., 2012). In the present study, there was no significant difference between the two groups for the death rate up to 2 weeks in the post-infarction period. A major factor is that cardiac rupture and arrhythmia caused a high rate of death during the first 2 weeks after surgery, while short-term orientin treatment could not reverse these complications. However, orientin reduced mortality 4 weeks after MI. Echocardiography is superior to invasive hemodynamics in evaluating cardiac function post-MI (Ishikawa et al., 2012). In our study, echocardiography and hemodynamics were both evaluated 4 weeks after MI. Orientin prevented post-infarction heart failure and ameliorated cardiac diastolic and systolic function. We have observed decreased cardiomyocyte apoptosis, cardiac fibrosis, and inflammatory response in the remodeling heart post-infarction after treatment with orientin. These data support the notion that orientin has a protective effect on cardiac remodeling post-infarction.

Reactive oxygen species negatively affects myocardial calcium handling, causes arrhythmia, and contributes to cardiac remodeling by inducing hypertrophic signaling, apoptosis, and necrosis (Munzel et al., 2017). In the failing heart, oxidative stress occurs in the myocardium and correlates with LV dysfunction (Tsutsui et al., 2011). Similarly, oxidative balance in the heart is tightly regulated by a wealth of pro- and antioxidant systems that orchestrate region-specific ROS production and removal (Robinson et al., 2011). Enzymatic sources for ROS, such as the NADPH oxidases (NOXs), uncoupled NO synthase, and mitochondria are all considered relevant sources of ROS in heart failure, causing vascular and myocardial dysfunction (Sawyer, 2011). The major defenses against ROS are antioxidative enzymes, including SOD, catalase, paraoxonases, glutathione peroxidase, and heme oxygenase (Sawyer, 2011). The imbalance of ROS and antioxidants contributes to the process of cardiac remodeling (Munzel et al., 2017). Previous study has reported that orientin inhibited high glucose-induced vascular inflammation and ROS generation (Ku et al., 2014). Orientin also alleviated cognitive deficits in a mouse model of Alzheimer’s disease by inhibition of oxidative stress (Yu et al., 2015). Consistently, our results revealed that orientin decreased the ROS generation and increased antioxidant activity and thus reduced the oxidative stress. In addition, ROS scavenger NAC could exert the same effects of orientin.

The mechanism by which orientin reduces oxidative stress is still unclear. NO, known as the “endothelium-derived relaxing factor” or “EDRF” is a small molecule and free radical. It is important to emphasize that NO has been shown to have a significant role in oxidation–reduction (Zhang and Casadei, 2012). NO can directly neutralize superoxide by quenching it, so reducing overall oxidative stress, which happens in absence of changes in protein or activity of NOS, particularly Enos (Paolocci et al., 2001). Heart failure is associated with decrease in NO bioavailability, which exerts deleterious effects accelerating heart failure progress (Wiemer et al., 2001). In our study, NO was decreased in remodeling heart post-infarction, while orientin increased NO production both in remodeling heart and hypoxia cardiomyocytes. NOSs are enzymes containing heme prosthetic groups that are crucial in the synthesis of NO from L-arginine. Three major types of NOS exist in humans: neuronal NOS (nNOS), cytokine-inducible NOS (iNOS), and endothelial NOS (eNOS) (Tang et al., 2014). eNOS is constitutively expressed in endothelium (Kuboki et al., 2000). Moreover, eNOS activation and NO production are already universally considered to be a cardioprotective mechanism (Heusch, 2015, 2017). The normal function of the vasculature needs the endothelium-derived eNSO/NO signaling (Cauwels et al., 2006). Recently, Thorsten et al. reported that endothelial-derived eNOS/NO in cardiomyocytes protected cardiomyocyte from I/R injury (Leucker et al., 2013). The present study showed that by activation of eNOS, orientin increased NO production in remodeling heart, which inhibited the excess oxidative stress, and contributed to the sustained cardio-protection. We observed that the cardio-protective effects of orientin disappeared after L-NAME but not L-VNIO treatment. Conversely, it seems that iNOS-derived NO causes negative effects during heart failure (Drexler et al., 1998). In our study, iNOS was observed increased in remodeling mice heart, but orientin did not affect the expression of iNOS. Moreover, the iNOS inhibitor L-Canavanine could not have abolished the protective effects of orientin. All these findings support the idea that orientin protects against cardiac remodeling via eNOS/NO signaling.

Previous studies have reported that orientin could protect against myocardium ischemic/reperfused injury via inhibition mitochondrial permeability transition (Lu et al., 2012) and apoptosis (Fu et al., 2006). These studies implicated that orientin may attenuate the ischemic injury, which leads to decreased MI size, causing the suppressed remodeling process. In this study, we observed decreased MI size and improved cardiac remodeling. Further study concerning whether orientin mitigates cardiac remodeling exclusively due to reducing the initial MI size or due to anti-remodeling activities is warranted.

## Conclusion

The current study demonstrates that orientin provides sustained cardio-protection against cardiac remodeling induced by MI. Importantly, the activation of eNOS/NO signaling facilitates the protective effects of orientin.

## Author Contributions

FL and WQ contributions to study conception and designed experiments. HZ, LX, KL, and LY carried out experiments. FL, JZ, and HY analyzed experimental results. FL, PZ, and JZ revised the manuscript. FL and WQ wrote and revised the manuscript.

## Conflict of Interest Statement

The authors declare that the research was conducted in the absence of any commercial or financial relationships that could be construed as a potential conflict of interest.

## Abbreviations

CTGF: connective tissue growth factor
dP/dt max: maximal rate of pressure development
dP/dt min: minimal rate of pressure decay
EF: ejection fraction
GAPDH: glyceraldehyde-3-phosphate dehydrogenase
H&E: hematoxylin and eosin
H/R: hypoxia/reoxygenation
HR: heart rate
I/R: ischemia/reperfusion
IL: interleukin
LV: left ventricle
LVEDd: LV end-diastolic diameter
LVESd: LV end-systolic diameter
LVPWd: LV posterior wall thickness
MHC: myosin heavy chain
MI: myocardial infarction
NO: nitric oxide
NRCM: neonatal rat cardiomyocyte
PSR: picrosirius red
ROS: reactive oxygen species
SOD: superoxide dismutase
TGFβ: transforming growth factor β
TNF: tumor necrosis factor
